# Immune Checkpoint Inhibitors in Advanced Prostate Cancer: Current Data and Future Perspectives

**DOI:** 10.3390/cancers14051245

**Published:** 2022-02-28

**Authors:** Sara Elena Rebuzzi, Pasquale Rescigno, Fabio Catalano, Veronica Mollica, Ursula Maria Vogl, Laura Marandino, Francesco Massari, Ricardo Pereira Mestre, Elisa Zanardi, Alessio Signori, Sebastiano Buti, Matteo Bauckneht, Silke Gillessen, Giuseppe Luigi Banna, Giuseppe Fornarini

**Affiliations:** 1 Medical Oncology Unit, Ospedale San Paolo, 17100 Savona, Italy; 2 Department of Internal Medicine and Medical Specialties (Di.M.I.), University of Genova, 16132 Genova, Italy; 3 Candiolo Cancer Institute, FPO-IRCCS, 10060 Candiolo, Italy; pasquale.rescigno@ircc.it (P.R.); gbanna@yahoo.com (G.L.B.); 4 Medical Oncology Unit, IRCCS Ospedale Policlinico San Martino, 16132 Genova, Italy; catalan.fab@gmail.com (F.C.); giuseppe.fornarini@hsanmartino.it (G.F.); 5 Medical Oncology, IRCCS Azienda Ospedaliero-Universitaria di Bologna, 40138 Bologna, Italy; veronica.mollica7@gmail.com (V.M.); francesco.massari@aosp.bo.it (F.M.); 6 Oncology Institute of Southern Switzerland (IOSI), Ente Ospedaliero Cantonale (EOC), 6500 Bellinzona, Switzerland; ursula.vogl@eoc.ch (U.M.V.); ricardo.pereiramestre@eoc.ch (R.P.M.); silke.gillessensommer@eoc.ch (S.G.); 7 Department of Medical Oncology, IRCCS Ospedale San Raffaele, Vita-Salute San Raffaele University, 20132 Milan, Italy; marandino.laura@hsr.it; 8 Institute of Oncology Research (IOR), 6500 Bellinzona, Switzerland; 9 Academic Unit of Medical Oncology, IRCCS Ospedale Policlinico San Martino, 16132 Genova, Italy; elisa.zanardi@hsanmartino.it; 10 Department of Health Sciences (DISSAL), University of Genova, 16132 Genova, Italy; alessio.signori.unige@gmail.com (A.S.); matteo.bauckneht@gmail.com (M.B.); 11 Medical Oncology Unit, University Hospital of Parma, 43100 Parma, Italy; sebabuti@libero.it; 12 Department of Medicine and Surgery, University of Parma, 43126 Parma, Italy; 13 Nuclear Medicine, IRCCS Ospedale Policlinico San Martino, 16132 Genova, Italy; 14 Faculty of Biomedical Sciences, Università della Svizzera Italiana (USI), 6900 Lugano, Switzerland; 15 Division of Cancer Sciences, University of Manchester, Manchester M13 9PL, UK

**Keywords:** prostate cancer, castration-sensitive, castration-resistant, immunotherapy, immune checkpoint inhibitor, predictive biomarkers, prognostic biomarkers, molecular oncology, tumor microenvironment

## Abstract

**Simple Summary:**

The treatment landscape of advanced prostate cancer (PCa) is constantly improving with the approval of many new therapeutic options. Immunotherapy in PCa has been investigated with disappointing results. This review aims to evaluate the potential role of immunotherapy in both castration-sensitive and castration-resistant PCa, discussing the immunobiology of PCa, the results of the current literature, and the ongoing clinical trials. Potential prognostic and/or predictive factors and future perspectives are also discussed.

**Abstract:**

In the last 10 years, many new therapeutic options have been approved in advanced prostate cancer (PCa) patients, granting a more prolonged survival in patients with metastatic disease, which, nevertheless, remains incurable. The emphasis on immune checkpoint inhibitors (ICIs) has led to many trials in this setting, with disappointing results until now. Therefore, we discuss the immunobiology of PCa, presenting ongoing trials and the available clinical data, to understand if immunotherapy could represent a valid option in this disease, and which subset of patients may be more likely to benefit. Current evidence suggests that the tumor microenvironment needs a qualitative rather than quantitative evaluation, along with the genomic determinants of prostate tumor cells. The prognostic or predictive value of immunotherapy biomarkers, such as PD-L1, TMB, or dMMR/MSI-high, needs further evaluation in PCa. Monotherapy with immune checkpoint inhibitors (ICIs) has been modestly effective. In contrast, combined strategies with other standard treatments (hormonal agents, chemotherapy, PARP inhibitors, radium-223, and TKIs) have shown some results. Immunotherapy should be better investigated in biomarker-selected patients, particularly with specific pathway aberrations (e.g., AR-V7 variant, HRD, CDK12 inactivated tumors, MSI-high tumors). Lastly, we present new possible targets in PCa that could potentially modulate the tumor microenvironment and improve antitumor activity with ICIs.

## 1. Introduction

Prostate cancer (PCa) is the most commonly diagnosed cancer and second most common cause of cancer-associated death (after lung cancer) among adult men [1]. Radical prostatectomy and radiotherapy with or without androgen-deprivation therapy (ADT) are the standard curative treatments for localized PCa [2,3], while ADT remains the mainstay therapy for advanced disease. Metastatic hormone-sensitive (mHSPC) and castration-resistant (mCRPC) PCa [2,3] represent, however, a major clinical challenge, and several types of treatments, including androgen receptor targeting inhibitors (ARTi), chemotherapies, radioligands, and PARP inhibitors, have been investigated to improve patient survival [4].

Immunotherapy has improved survival and response outcomes with a manageable safety profile in many advanced malignancies, while its efficacy has been modest in patients with PCa, to date [5]. Indeed, other than sipuleucel-T, the first Food and Drug Administration (FDA)-approved autologous cellular therapeutic vaccine, no other immunotherapeutic strategies have been registered for the treatment of advanced PCa, due to their limited response rates and modest clinical efficacy in unselected patients [6]. However, advancements in our understanding of the molecular pathophysiology of cancer immune response and PCa might lead to a shift in the paradigm of immunotherapy for the treatment of PCa.

This review aims to describe the role of immunotherapy in advanced PCa, evaluating the biologic rationale of using this treatment modality, describing the preliminary results published and the ongoing trials.

## 2. Immunobiology of Prostate Cancer

**Figure 1:**
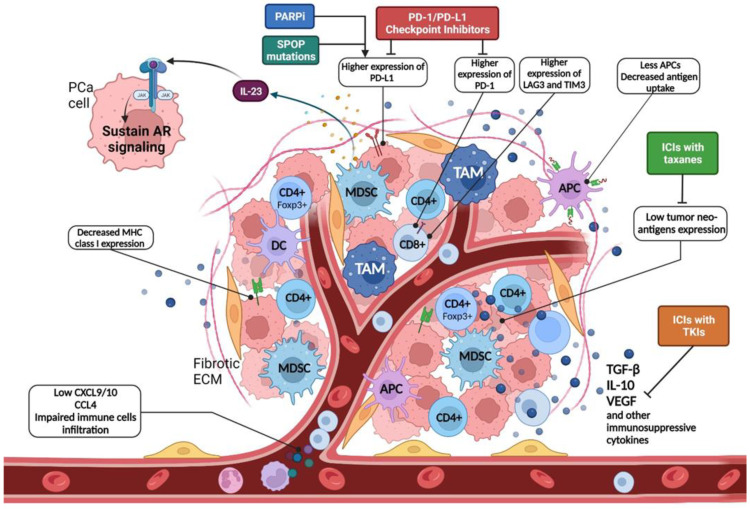
Immunosuppressive tumor microenvironment and how we can overcome it. PARP-i poly(ADP-ribose) polymerase inhibitor, PD-1 programmed cell death-1, PD-L1 programmed cell death ligand 1, SPOP speckle type BTB/POZ protein, IL interleukin, LAG3 lymphocyte activating 3, TIM3 T-cell immunoglobulin domain and mucin domain 3, APC antigen-presenting cell, PCa prostate cancer, AR androgen receptor, ICIs immune checkpoint inhibitors, TAM tumor-associated macrophage, MDSC myeloid-derived suppressor cell, DC dendritic cell, MHC major histocompatibility complex, TKIs tyrosine kinase inhibitors, ECM extracellular matrix, TGF transforming growth factor, VEGF vascular endothelial growth factor, CXCL chemokine (C–X–C motif) ligand, CCL chemokine ligand.

PCa is generally considered immunologically ‘cold’, characterized by T-cell exclusion, low neoantigen load, and a relative highly immunosuppressive microenvironment [7,8] (Figure 1). 

Furthermore, the immune cells that are part of the prostate tumor microenvironment are frequently characterized by an anergic and immunosuppressive phenotype and include regulatory T-cells (Tregs), M2-polarized tumor-associated macrophages (TAMs), and myeloid-derived suppressor cells (MDSCs) [8]. Specifically, MDSCs are a heterogeneous population of activated immune cells expanded in pathological conditions, including cancer, with potent immunosuppressing activity and a potential role in PCa endocrine resistance [9]. Previous data demonstrated that MDSCs could support tumorigenesis in many cancer types through different mechanisms, with preclinical studies indicating that the inactivation of MDSCs increased immune checkpoint blockade efficacy in CRPC models [10,11]. Castration-resistant PCa is enriched in MDSCs, and interleukin-23 (IL-23) produced by MDSCs can regulate castration resistance by sustaining androgen receptor (AR) signaling [10]. Indeed, inhibition of IL-23 can reverse ADT resistance in mouse models. Therefore, this could represent a possible target in men suffering from advanced PCa [10] and is currently being tested in clinical trials.

The tumor microenvironment is a pivotal factor that influences therapy responses in multiple ways [12]. Tumor-infiltrating lymphocytes (TILs) play a central role and have been demonstrated to be prognostic indicators of a better outcome in colorectal cancer, melanoma, and breast cancer [12,13,14]. In PCa patients, their prognostic role remains controversial [15,16] As already pointed out, PCa presents a low immunogenicity with few TILs in the tumor microenvironment (100 CD8^+^ T cells per mm^2^) [17,18]. This limited presence of TILs translates into a scant adaptive immune response against tumor cells. Furthermore, a significant part of TILs in mCRPC resulted to be CD4^+^ regulatory T cells (Tregs), while CD8^+^ cells present an exhausted phenotype with higher expression of inhibitory factors, such as PD1, LAG3, and TIM3 [19].

Moreover, PCa tumor cells frequently present PTEN loss (20% in primary tumors, 40% of mCRPC patients), which interacts with the interferon-1 (IFN-1) pathway, a pivotal step in the immune response, leading to its dysfunction and immunosuppression [20,21]. In fact, it has been shown that PTEN knockout cells have decreased IFN type I response due to a dysfunctional nuclear import and lowered interferon regulatory factor 3 (IRF3) gene activity [22]. Furthermore, PTEN-null senescent tumors released immunosuppressive cytokines with the activation of the Jak2/Stat3 pathway and consequently decreased the immune response in the tumor microenvironment [23].

Another factor that could influence the immune response is the presence of speckle-type pox virus and zinc finger protein (SPOP) missense mutations that occur in 10–15% of PCa patients [24]. These alterations have been associated with higher PD-L1 expression by compromising its ubiquitination-mediated degradation, thus leading to a more immunosuppressive microenvironment and making these tumors more likely responsive to anti-PD-L1 immunotherapy [23].

More interestingly, the TME of specific metastatic sites can confer immunosuppressive characteristics, as recently shown. PCa cells are indeed able to promote the expression of bone-specific markers, a phenomenon known as osteomimicry. This allows tumor cells to escape the immune detection within the bone TME, increasing their chance of survival [25].

Using digital spatial profiling (DSP) technologies, Bradly and colleagues performed transcriptomic and protein expression studies in spatially distinct regions of mPC biopsies [18]. They found a high level of intra-patient homogeneity relative to tumor characteristics such as AR and neuroendocrine activities. In this work, they were able to assess that, while the majority of metastases do not present significant inflammatory infiltrates and lack PD1, PD-L1, and CTLA4 (targets recently evaluated in clinical trials), the B7-H3/CD276 immune checkpoint protein was highly expressed, particularly in those cancers with high AR activity [18].

A subset of lethal PCas (20–30%), including those with defective homologous recombination DNA repair genes, defective mismatch repair (dMMR) genes, DNA polymerase epsilon (POLE) mutations, and cyclin-dependent kinase 12 (CDK12) biallelic alterations, have been variably associated with higher tumor mutational burden (TMB) and neoantigen load, which may arguably increase the likelihood of antitumor immunity [7,26].

Moreover, cancer genomic determinants have also been associated with different immunological profiles. More specifically, analyzing genomic and transcriptomic data from the PCF/SU2C cohort, Rodrigues and colleagues demonstrated that dMMR mutational signatures were associated with increased T-cell related transcripts, as well as immune checkpoint-related transcripts, including PD-L1 and PD-L2. These data have also been confirmed through IHC studies on dMMR tumor tissues [17]. Moreover, dMMR tumors also present higher expression of the metabolic immune checkpoint adenosine receptor 2A (ADORA2A) and a prominent expression of markers attributable to myelomonocytic cells (i.e., VCAM1, NLRP3, and JAK2) described to mediate MDCS expansion and accumulation [17]. These data justify combination therapies with ICIs in dMMR mPC [27].

Similarly, CDK12 biallelic loss PCa seems to be characterized by an increased CD3 density compared to PC without this genomic aberration. However, when qualitatively investigated, the TME of CDK12 altered tumors seems to comprise CD4^+^Foxp3^+^, associated with poorer survival [28].

These data would indicate that, beyond the cold/hot tumor dichotomy, the TME in mCRPC needs a qualitative rather than quantitative evaluation, along with the genomic determinants of prostate tumor cells [29].

In this context, a patient’s selection for immunotherapy can be the key to better identifying who would potentially benefit from this approach.

## 3. Immune Checkpoint Inhibitors in Hormone-Sensitive Prostate Cancer

### 3.1. Current Evidence

As previously mentioned, ICIs have not shown promising results in PCa in unselected patients, neither in the mCRPC setting nor in earlier stages of the disease in the hormone-sensitive setting where only very scarce data exist. In this setting, the only published study is a single-arm, single-institution, pilot trial on cryotherapy to the prostate gland combined with pembrolizumab and ADT in oligometastatic HSPC patients [30]. The primary study endpoint was the percentage of patients at 1 year with a PSA < 0.06 ng/mL and the number and grade of adverse events (AEs). The rationale to combine radiotherapy (RT) or local ablative treatments such as cryoablation with an ICI stands in an expected enhanced antigen release caused by the RT, leading to a potential increased response to checkpoint inhibition. Twelve patients were enrolled, and 42% of them presented with a PSA level under the predefined threshold at 1 year, and no grade 3 adverse events (AEs) or higher were reported. Local control of the prostate tumor was encouraging, although systemic control after testosterone recovery was not maintained (Table 1) [30].

### 3.2. Ongoing Trials

Phase Ib to phase III trials on ICIs in combination with approved or other experimental drugs in mHSPC patients (Table 1) are ongoing, and results are awaited in the future.

The largest ongoing phase III trial in mHSPC patients is the KEYNOTE-991 trial, planned to enroll over 1000 patients who are treatment-naïve or have received a maximum of six cycles of docetaxel. Patients are randomized to pembrolizumab or placebo plus enzalutamide and ADT with radiographic progression-free survival (rPFS) and overall survival (OS) as coprimary endpoints.

Other earlier phase and smaller trials in mHSPC patients include chemo-immunotherapy strategies with the combinations of docetaxel + ADT with nivolumab and ipilimumab (NCT03879122) or with cemiplimab (NCT03951831).

A phase Ib trial CABIOS (NCT04477512) integrates the multi-tyrosine kinase inhibitor cabozantinib and abiraterone/prednisone in addition to nivolumab.

The phase II POSTCARD trial (NCT03795207) evaluates the immune response generated by radiotherapy and ICIs in PET-PSMA-positive oligometastatic patients who are negative for metastases on conventional imaging. This trial hypothesizes that the PDL-1 inhibitor durvalumab will enhance immune response following SBRT targeting oligometastatic lesions. Durvalumab is started 1 month before SBRT (total of 12 months) to evaluate PSA response to immunotherapy alone. Of note, this trial does not contemplate the addition of ADT to the experimental treatments.

A phase II trial (NCT04126070) is exploring ADT plus nivolumab plus docetaxel in three different cohorts with either DNA damage repair (DDR) defects or inflamed tumors or without those characteristics.

## 4. Immune Checkpoint Inhibitors in Castration-Resistant Prostate Cancer

### 4.1. Current Evidence: Monotherapy

Several clinical trials investigated the use of ICIs in patients with mCRPC. In 2014, Kwon et al. reported the results of a phase III randomized trial in 799 mCRPC patients with at least one bone metastasis who progressed after docetaxel and were randomly assigned to bone-directed radiotherapy followed by either ipilimumab or placebo [31]. The primary analysis did not show statistically significant differences in terms of OS in the two treatment arms (median overall survival—mOS: 11.2 vs. 10, hazard ratio—HR 0.85, *p* = 0.053), but the HR changed from 1.46 in the first 5 months to 0.60 after 12 months of therapy suggesting promising results in the long-term period with immunotherapy [31]. Moreover, the final analysis after a follow-up of 2.4 years showed a survival benefit 2–3 times higher in the ipilimumab cohort [32].

The use of anti-CTLA4 antibody ipilimumab was also investigated in chemo-naïve mCRPC patients. In a phase III randomized trial, mCRPC patients were randomly assigned to receive placebo or ipilimumab 10 mg/kg every 3 weeks up to four cycles and a maintenance therapy with ipilimumab 10 mg/kg every 3 months [33]. No survival benefit was observed (mOS 28.7 vs. 29.7, HR 1.1, *p* = 0.37), but increased median progression-free survival (mPFS) and PSA response rate (RR) were reported in the immunotherapy arm [33].

Both studies with ipilimumab in patients with mCRPC showed no benefit in OS. Nevertheless, promising results were observed in the long-term period and in terms of antitumor activity in a subset of patients.

Preliminary results were also reported with pembrolizumab in mCRPC patients. The KEYNOTE-028 study was a phase Ib trial on pembrolizumab in patients with advanced solid tumors, including heavily pretreated mCRPC with PD-L1 ≥ 1% using combined score with Dako assay test (~70% of patients received at least two prior systemic therapies) [34]. In the cohort of mCRPC patients (*n* = 23), pembrolizumab showed preliminary evidence of antitumor activity with a favorable safety profile [34].

In the phase II KEYNOTE-199 study, the activity and safety of pembrolizumab were assessed in a large group of patients with pretreated mCRPC divided into three cohorts: cohort 1 (*n* = 133) included patients with PD-L1-positive disease, cohort 2 (*n* = 66) included patients with PD-L1-negative disease, and cohort 3 (*n* = 59) enrolled patients with bone predominant disease, regardless of PD-L1 expression [35]. Median OS and disease control rate (DCR) were higher in cohort 3 patients compared to cohorts 1 and 2. Overall response rate (ORR) was 3% in cohort 2 and 5% in cohort 1, respectively, while PSA response rate was 6% in cohort 1, 8% in cohort 2, and 2% in cohort 3. Authors concluded that pembrolizumab showed antitumor activity in certain groups of patients (bone-predominant mCRPC), previously treated with docetaxel and endocrine therapy [35].

In heavily pretreated patients, atezolizumab monotherapy demonstrated evidence of disease control with a 12 month OS rate of 52.3%, in this setting, albeit with a minimal response rate (confirmed 50% PSA response rate was 8.6%, 3/35 patients) [36].

### 4.2. Current Evidence: Combination Therapies

Given the modest results of anti-PD1/PD-L1 and anti-CTLA4 antibodies as monotherapy in patients with mCRPC, several immunotherapy-based combinations have been investigated.

The phase III IMbassador 250 trial was the first study to investigate an immunotherapy combination with atezolizumab plus enzalutamide vs. enzalutamide alone in 759 mCRPC patients who progressed on abiraterone and were ineligible for or refused a taxane regimen [37]. This study did not meet the primary endpoint of improved overall survival in unselected patients (stratified HR 1.12, 95% CI 0.91–1.37, *p* = 0.28). However, the extensive biomarker analyses associated with this study support further validation of predictive biomarkers to ICIs in mCRPC. More specifically, approximately 2.9% of patients (22 of 759) had tumors that exhibited PD-L1 expression on the immune component of ≥5% to <10%, and ≥10% (IC2/3, VENTANA SP142, Roche Diagnostics). Fewer PFS events were observed in patients with IC2/3 tumors in the combination arm (HR 0.28; 95% CI 0.12–0.66), and those tumors with CD8 infiltration above the mean (HR 0.72; 95% CI 0.54–0.96). Unfortunately, the genomic analysis did not detect CDK12 biallelic alterations, and the only two MSI-high identified patients did receive enzalutamide alone. No immunohistochemical assays to detect protein expression of MMR were performed in this study. RNA sequencing data confirmed the relevant information leveraged from transcriptomic analyses, showing that expression of genes within immune-related pathways, including IFN and PD-1 signaling, was associated with longer PFS in the atezolizumab + enzalutamide arm [37].

The single-arm phase II CheckMate 650 trial investigated the immune combination nivolumab + ipilimumab for four cycles followed by nivolumab as maintenance therapy in patients with mCRPC divided into two cohorts: cohort A with symptomatic or minimally symptomatic patients progressed after ≥1 s generation hormone therapy (chemo-naïve) and cohort B with patients progressed after chemotherapy [38]. The preliminary results on 90 patients showed higher ORR (25 vs. 10%), mPFS (5.5 vs. 3.8 months), and mOS (19.0 vs. 15.2 months) in chemo-naïve patients (cohort A, *n* = 45) compared to patients progressed to chemotherapy (cohort B, *n* = 45). However, several grade 3–4 toxicities were reported; thus, dose/schedule modification is needed to optimize safety [38].

The combination therapy with nivolumab + ipilimumab has also been investigated in the phase II STARVE-PC trial in patients with AR-V7-expressing mCRPC divided into two cohorts: cohort 1 patients receiving nivolumab + ipilimumab alone and cohort 2 receiving nivolumab + ipilimumab + enzalutamide [39]. Efficacy results were not statistically different in the two cohorts of patients; hence, the authors concluded that the immune combination of nivolumab + ipilimumab showed only modest activity in mCRPC patients with AR-V7 variant [39].

The multi-arm randomized phase II trial CheckMate 9KD study investigated nivolumab in combination with other therapies such as rucaparib (Arm A), docetaxel (Arm B), or enzalutamide (Arm C) in mCRPC patients who were differently pretreated [40]. Preliminary results of Arm B were reported at the 2021 ASCO GU Cancer Symposium (complete data yet to be published); the combination of nivolumab + docetaxel showed encouraging clinical activity in terms of survival and response outcomes [40]. The final results for mCRPC patients receiving nivolumab + rucaparib post chemotherapy (Arm A1) were reported at the 2021 ASCO Annual Meeting; the combination was active in HRD-positive patients, while HRD-negative patients did not appear to benefit from this combination therapy [40]. The final analysis of the arm A2 cohort on 71 mCRPC patients treated with nivolumab + rucaparib after 1–2 prior lines of novel hormonal therapy (chemo-naïve) was presented at the ESMO 2021 congress [41]. It showed a better response (ORR, PSA-RR) and survival outcomes (rPFS, mOS) in HRD-positive mCRPC patients vs. HRD-negative/not evaluable tumors [41].

### 4.3. Ongoing Trials

The ongoing clinical trials on immunotherapy in mCRPC are collected in Table 2. The principal primary endpoints regard response outcomes (i.e., ORR, PSA decline) and safety profile.

Most studies are investigating the use of ICI as monotherapy in the subgroup of mCRPC patients harboring different types of gene mutations, which have been suggested to be associated with a better response to immunotherapy. Pembrolizumab is being tested after one prior line of treatment in mCRPC patients with CDK12 biallelic inactivation (NCT04104893) or with high mutation load and/or DNA repair deficiency (PERSEUS 1, NCT03506997). Furthermore, combination therapy with nivolumab + ipilimumab is currently being investigated in patients with an “immunogenic” phenotype (dMMR and/or a high TMB of >7 mutations per Mb BRCA2 inactivation or BRCAness signature; tandem duplication signature and/or CDK12 biallelic inactivation) (INSPIRE, NCT04717154) or CDK12 mutation (IMPACT, NCT0357619).

Some clinical trials are investigating the combination of ICI with standard treatment for mCRPC patients not selected for genomic signatures or mutations, including the combination of nivolumab with radium-223 in the Rad2Nivo trial (NCT04109729) and with docetaxel in the phase III Checkmate 7DX trial (NCT04100018).

Several studies are also investigating combination therapy with ICI with tyrosine kinase inhibitors (TKI) in patients with mCRPC progressed to second-generation hormonal therapy and/or chemotherapy. The phase II NCT04159896 trial assesses the combination of ESK981, a pan-VEGFR/TIE2 TKI, with nivolumab, while the CONTACT-02 trial (NCT04446117) is a phase III trial investigating the combination of atezolizumab plus cabozantinib versus abiraterone + prednisone or enzalutamide (after having failed on the alternate drug).

Other studies are assessing the activity of new drugs in combination with ICI and/or standard therapies (ARTi, chemotherapy or radiometabolic therapy). Among those, there are acapatamab (AMG160), a PSM—bispecific T-cell engager (NCT04631601, NCT03792841), M7824, a bifunctional fusion protein targeting TGF-β and PD-L1 (NCT04633252), and W_pro1 (BNT112), an mRNA targeting five antigens expressed in PCa cells (PRO-MERIT trial, NCT04382898).

## 5. Clinical Trial on Molecularly Selected PCa Patients

### 5.1. Molecular Pathways

The development of ICIs in solid tumors is accompanied by the clinical need to identify prognostic and predictive biomarkers that could help in the decision-making process. Among these, the most studied in different tumor types are high programmed death receptor-ligand 1 (PD-L1), microsatellite instability (MSI) or dMMR, high TMB, DNA damage repair (DDR) defects, and factors correlated to the microenvironment.

### 5.2. PD-L1 Expression

PD-L1 expression has been evaluated as a predictive biomarker of response to ICIs in multiple tumor histologies, such as head and neck, non-small-cell lung, and gastric cancer [42]. In other tumor types, it was not predictive of response to immunotherapy, showing only a prognostic value, such as in renal cell carcinoma. Furthermore, the PD-L1 positivity cutoff value and evaluation method vary among different tumor types. Regarding PCa, several studies investigated the prognostic and predictive role of PD-L1. PD-L1 expression seems to be high in both primary tumors (about 20%) and mCRPC stage (up to 90%) [43]. Its expression does not seem to predict response to ICIs [44]. On the other hand, the negative prognostic value of PD-L1 expression has been highlighted in several studies [45]. In particular, high PD-L1 expression was associated with a higher risk of recurrence after radical prostatectomy [45].

### 5.3. DNA Damage Repair Genes

DDR gene alterations have been observed in 23% of mCRPCs [46]. The most frequent mutations are found on BRCA2, ATM, CHEK2, and BRCA1 genes [47]. The presence of alterations in these genes might increase the DNA mutational load and, consequently, genomic instability, resulting in an enhanced antitumor immune response [48]. Furthermore, BRCA2 mutation is associated with more aggressive behavior, characterized by frequent lymph node and distant site involvement. A novel therapeutic approach entering into the clinical scenario of PCa patients is poly(adenosine diphosphate-ribose) polymerase (PARP) inhibition. PARP inhibitors interact with the DDR gene pathway by blocking the DNA repair gene mechanism, especially in patients with other DDR gene alterations (such as BRCA1 or 2 or ATM mutation), leading to cancer cell death [49]. Moreover, PARP inhibition leads to increased PD-L1 expression, a higher release of neoantigens and consequent increased immune response, paving the way to exploring a combination of PARP inhibition and immunotherapy [50].

Among DDR gene alterations, CDK12 deserves a separate mention since it has been associated with distinct behavior from other mutations. In fact, CDK12-mutated PCas are typically linked with a poor prognosis and do not present a markedly high sensitivity to PARP inhibitor monotherapy but, as previously mentioned, present increased neoantigen load and lymphocytic infiltration [51]. These characteristics might make CDK12-altered tumors more responsive to anti-PD1, as evidenced in retrospective case report studies in which patients with CDK12 mutation treated with nivolumab or pembrolizumab presented a 33% PSA response and median PFS of 5.4 months [51].

### 5.4. Tumor Mutational Burden

Another biomarker with great relevance in the immunotherapy field is TMB. It expresses the number of mutations per tumor or megabase of DNA sequenced (mut/Mb) and could reflect immunogenic neoantigens. A high TMB, consisting of ≥10 mut/Mb, has been correlated to enhanced responses to ICIs [52,53]. The individuation of TMB as a predictive biomarker has led the FDA to approve pembrolizumab in all solid tumors with a TMB ≥ 10 mut/Mb, considering the durable responses evidenced in the KEYNOTE-158 phase II study [54]. Nonetheless, among the limitations of this study, it has to be underlined that, unlike colon cancer, only a small subset of PCa patients present with unstable microsatellite cancers [17]. Furthermore, a recently published work analyzing over 10,000 tumors from The Cancer Genome Atlas showed that, in PCa, breast cancer, and glioma, there was no correlation between CD8^+^ T-cell expression and neoantigen load, and TMB was not predictive of response to ICI [54]. This is probably due to the immunologically cold behavior of PCa, which results in a low attraction of T cells into the tumor microenvironment [55].

All these factors make PCa generally resistant to immunotherapy approaches, thus leading to the investigation of combination approaches, such as the addition of PARP inhibitors, chemotherapy, or ARTi, which could help to enhance antitumor immune response.

### 5.5. Mismatch Repair Pathway/Microsatellite Instability

The mismatch repair (MMR) pathway is a DNA repair mechanism specific to replication errors, including mismatch base-pairing and nucleotide insertions and deletions [56,57]. The MMR pathway plays a crucial role in maintaining genomic stability [58], and defective MMR results in MSI and a hypermutator phenotype [17].

The Food and Drug Administration tissue-agnostic approval of the PD-1 inhibitor pembrolizumab for the treatment of dMMR or MSI-high (MSI-H) cancers in 2017 led to a renewed interest in exploring alterations of this DNA repair system to identify those patients most likely to benefit from immunotherapy [59].

Considering the low prevalence of dMMR or MSI-H PCa, ranging from 3% to 22% in various studies [17,59,60,61,62,63], comprehending the potential prognostic or predictive role of MMR pathway alterations is challenging. Moreover, the optimal method for determining MSI-H/dMMR status in PCa is still debated [60].

Even if the evidence is limited due to the paucity of published data, various studies suggested a negative prognostic role for dMMR pathway, which may confer an aggressive phenotype, possibly related to the polyclonal, highly dynamic genomic landscape of these tumors [17,59,61,64]. The retrospective study by Graham et al., including 27 consecutive men with dMMR or MSI-high metastatic PCa from two academic institutions, showed evidence of aggressive clinical and pathological features, with a high rate of de novo metastatic PCa (48% of patients) and a high incidence of Gleason score ≥8 (79% of patients) [61]. These results follow previous retrospective data by Antonarakis et al., showing 46% of patients with dMMR tumors having metastatic disease at diagnosis, 31% visceral metastases, and 75% grade group 5 (Gleason score 9 or 10) [59]. To further support the possible negative prognostic value of dMMR, Rodrigues et al. found that evidence of dMMR was associated with decreased OS in a cohort of approximately 120 patients with non-indolent PCa [17].

Data regarding the response of dMMR PCa to standard treatment are conflicting. Some studies reported response and survival outcomes to standard therapies, which were similar to those reported in unselected patients [59,61], while other studies showed poor response to hormonal treatments in dMMR PCa patients [60,63]. Considering the low number of patients included in these studies and the different methods used to assess MMR/MSI status, no firm conclusion can be drawn regarding the response to standard treatments of dMMR PCa.

Little literature exists on patients with dMMR PCa treated with immunotherapy. Abida et al. reported a PSA50 response rate of 54.5% in 11 patients with dMMR/MSI-H PCa with a durable response, and four of these patients also had a radiographic response [60]. A PSA50 response of 50% was also found by Antonarakis et al. in four patients with MMR-mutated advanced PCa treated with anti-PD1 immunotherapy (pembrolizumab or nivolumab) [59]. Graham et al. reported a similar PSA50 response rate of 53%, with 87.5% of patients with a PSA50 response being progression-free at a median follow-up of 12 months, and a PFS at 6 months of 64.1% in 17 patients with dMMR and/or MSI-H metastatic PCa [61]. Overall, these studies suggest that not all patients with dMMR respond to immunotherapy.

Rodrigues et al. found a higher likelihood of PD-L1 positivity in dMMR mCRPC, as 5/10 (50%) mCRPC samples were PD-L1 positive at the immunohistochemistry evaluation with a validated antibody to the PD-L1 carboxy-terminal domain, compared to 4/91 (9.8%) with pMMR [17]. However, even if associated with PD-L1 expression, T-cell infiltration was heterogeneous in mCRPC samples, suggesting that not all patients with dMMR mCRPCs have a higher density of TILs. A high expression of factors involved in T and natural killer (NK) cell recruitment (CCR and CXCRs) and function (PRF1, BTLA, and TNFRSF9/CD137) was found in dMMR tumors. However, markers related to myelomonocytic cells and MDSCs (i.e., VCAM1, NLRP3, JAK2) were also highly expressed in patients with dMMR, as well as CD36, a protein involved in M2 macrophage activation, and PI3Kγ, which is suggested to be involved in the immunomodulatory activity of tumor-associated myeloid subsets. These data suggest that combination treatments, aimed at targeting myeloid suppressor cells, may enhance the efficacy of immunotherapy in some patients with dMMR mCRPC [17].

On the basis of these preliminary results, many trials have been designed to demonstrate the efficacy of ICIs in dMMR/DDRD/high TMB cancers (Table 2).

In the phase II trial evaluating the combination of nivolumab and ipilimumab in PCa with an immunogenic signature (cohort 1 of the NEPTUNES multi-center trial), Linch and colleagues showed that 50/211 prescreened patients were biomarker-positive, of whom 35 patients were treated with the combination nivolumab/ipilimumab with a median follow-up of 7.2 months [65]. Overall response rate was 26% (*n* = 9/35), of which four had dMMR (*n* = 4/5), three had BRCA1/2 (*n* = 3/4), two had exclusively high TILs (*n* = 2/9), and one was CDK12-defective (*n* = 1/7).

## 6. Future Perspectives

The role of TME in PCa was described in the previous sections; here, we speculate the possibility of using the modulation of TME as a therapeutic target in conjunction with ICI strategies.

Cytokines, particularly interleukins, are attractive prognostic targets in human cancer, including PCa. Circulating levels of interleukin 6 (IL-6) and 8 (IL-8) have been associated with poor outcomes in metastatic prostate cancer patients [66,67].

Inhibitors of the IL-8 receptor, CXCR-2, have been investigated in mCRPC patients (identifier: NCT03177187) and could be combined with ICIs in future trials.

Similarly, IL-23, produced by MDSCs, has been linked to the emergence of the castration resistance status through the activation of AR pathway and could promote tumor progression by regulating the Th17 response and Treg functions [68], suggesting a role for its blockade in this clinical setting. Anti-IL-23 therapy, such as guselkumab, was recently approved by the FDA to treat psoriatic arthritis, and its effectiveness could also be explored in PCa [69].

An emerging biomarker is the ectoenzyme CD38, usually expressed in tumor and immune cells. CD38 is involved in the synthesis of adenosine, which can inhibit antitumoral immunity after interacting with its receptor on immune cells [29,70]. Recently, CD38^+^ tumor-infiltrating immune cells were identified in CRPCA and were correlated to worse OS [71]. Daratumumab, an anti-CD38 monoclonal antibody, is currently approved for heavily pretreated relapsed and refractory multiple myeloma patients with a good toxicity profile, representing a new possible partner for ICI strategies [72].

## 7. Conclusions

Immunotherapy-based approaches have enriched the therapeutical opportunities of many cancer types, improving patient survival; however, to date, in advanced PCa patients, it has no established role outside of retrospective small series data on dMMR/MSI high and CDK12 patients [60]. However, a biomarker suite predictive of response to ICIs has not been prospectively validated in ad hoc studies.

Very few data are currently available on the use of ICIs in mHSPC patients [2]. In this context, many ongoing trials are exploring the combination of immunotherapy with different drugs (hormonal agents, chemotherapy, and/or TKI) (Table 1). In the mCRPC setting, ICIs have been tested for longer, and we do have more clinical data available. However, no consistent benefits in terms of OS have been reported, whether with anti-PD1 or anti-CTLA4 ICIs in monotherapy. Due to the unsatisfactory results in monotherapy, several studies have assessed the combination of ICI and standard treatments (ARTi, chemotherapy, PARP inhibitors, radium-223, and TKIs), with promising results, especially in those patients with specific pathway aberrations (e.g., AR-V7 variant, HRD). However, it remains challenging to discriminate the actual impact of a single drug in such combinations without a comparator arm. Given these results, several clinical trials are specifically investigating the efficacy of ICI in mCRPC biomarker-selected patients, such as HRD-positive tumors, CDK12-inactivated tumors, MSI-high tumors, and several others, which might represent promising predictive and prognostic factors (Table 2) able to offer a more accurate patient selection in the rapidly evolving therapeutic scenario of advanced PCa. Nevertheless, considering the role of TME in response to ICIs, a bigger effort should be made in designing combinational trials investigating ICIs with immune-modulating agents and exploring the role of immunosuppression beyond PD1/PD-L1.

## Figures and Tables

**Table 1:** Clinical trials on ICIs in locally advanced or metastatic HSPC patients.

Available Clinical Trials	Number of Patients Patients’ Characteristics	Pretreatment	Study Interventional Method/Drug	Primary Endpoint	Results
Single-arm, single-institution pilot trial [30]	*n* = 12 Oligometastatic patients (≤5 extra-pelvic metastases)	Treatment-naïve	Whole prostate cryoablation + short-term ADT (8 months) + pembrolizumab (6 cycles)	Number of patients with 1 y PSA < 0.6 ng/mL Frequency of AEs	1 y-PSA < 0.6 ng/mL: 42% mPFS: 14 months mSTFS: 17.5 months AEs grade ≤ 2: 100%
**Ongoing Clinical Trials (Name/NCT, Phase)**	**Planned Number of Patients** **Patients’ Characteristics**	**Pretreatment**	**Study Interventional Method/Drug**	**Primary Endpoint**	**Estimated Primary Completion Date (Month/Year)**
PROSTRATEGY NCT03879122 Phase II/III	*n* = 135 High-volume disease (CHAARTED criteria)	Treatment-naïve (ADT < 120 days allowed)	Control arm: ADT + docetaxel (6 cycles) Experimental arm 1: ADT + docetaxel + nivolumab Experimental arm 2: ADT + ipilimumab alternating with docetaxel Experimental arm 3: ADT + ipilimumab alternating with docetaxel and with nivolumab	OS	July 2022
KEYNOTE-991 NCT04191096 Phase III	*n* = 1232 At least 2 bone lesions +/− visceral disease	Treatment-naïve (6 cycles docetaxel allowed)	Experimental arm: ADT + enzalutamide + pembrolizumab Control arm: ADT + enzalutamide + placebo	rPFS OS	July 2026
CABIOS NCT04477512 Phase Ib	*n* = 22	Treatment-naïve	Experimental level 1: cabozantinib 20 mg + abiraterone acetate + nivolumab Experimental level 2: cabozantinib 40 mg + abiraterone acetate + nivolumab Experimental expansion: cabozantinib + abiraterone acetate + nivolumab	DLTs	January 2022
REGN2810 NCT03951831 Phase II	*n* = 20	Treatment-naïve	ADT + cemiplimab + docetaxel (max 6 cycles)	Percentage of subjects achieving undetectable PSA at 6 months after combination treatment	September 2020
NCT04126070 Phase II	*n* = 60 Cohort 1: somatic or germline homozygous deletions and/or deleterious mutations in a DDR gene, MMRd, or MSI-H Cohort 2: PD-L1 positive and/or CD8^+^ T cell inflamed using ImmunoProfile without the presence of DDRD Cohort 3: negative for DDRD and PD-L1 with low CD8^+^ T-cell infiltration	Treatment-naïve	ADT + nivolumab + docetaxel (max 6 cycles)	Number of patients 1 y PSA ≤ 0.2 ng/mL	June 2023
POSTCARD NCT03795207 Phase II	*n* = 96 Oligometatastatic disease (≤5 bone/lymph node metastases detected only on FCH-PET/CT or Ga-PSMA PET/CT)	Biochemical recurrence after RT or RP	Experimental arm: SBRT + durvalumab (1 year) Control arm: SBRT 3 fractions (32 patients will be enrolled in this arm)	2 y PFS	November 2023

N: number of patients, ADT: androgen-deprivation therapy, PSA: prostate-specific antigen, 1 y PSA: prostate-specific antigen at 1 year, AEs: adverse events, mPFS: median progression-free survival, mSTFS: median systemic therapy-free survival, OS: overall survival, rPFS: radiological progression-free survival, DLTs: dose-limiting toxicities, DDRD: DNA damage repair defects, MMRd: mismatch repair deficiency, MSI-H: microsatellite instability high, PD-L1: programmed death-ligand 1, FCH-PET/CT: ^18^F-fluorocholine positron emission tomography/computed tomography, PSMA PET/TC: prostate-specific membrane antigen positron emission tomography/computed tomography, RT: radiotherapy, RP: radical prostatectomy, SBRT: stereotactic body radiation therapy, 2 y PFS: prostate-specific antigen at 2 years.

**Table 2:** Ongoing clinical trials on ICIs in mCRPC patients.

Clinical Trial (Name, NCT, Phase)	Planned Number of Patients Patients’ Characteristics	Pretreatment	Study Drug	Primary Endpoint	Estimated Completion Date
**Molecular-Selected Patients**					
CHOMP trial NCT04104893 Phase II	*n* = 30 MMD or somatic biallelic inactivation of CDK12	One 2nd generation hormonal therapy for mCSPC, M0CRPC and/or mCRPC setting (i.e., abiraterone acetate, enzalutamide, apalutamide or darolutamide)	Pembrolizumab	PSA50 ORR	March 2023
PERSEUS1 NCT03506997 Phase II	*n* = 100 High mutational load (≥11 mutations per targeted panel) on NGS and/or DNA repair defect including MMD	≥1 approved treatment for mCRPC (i.e., abiraterone acetate, enzalutamide, docetaxel, cabazitaxel, radium-233)	Pembrolizumab	PSA50 ORR	September 2023
INSPIRE NCT04717154 Phase II	*n* = 75 Immunogenic phenotype: MMD and/or high TMB (>7 mutations/Mb (cluster A); BRCA2 inactivation or BRCAness signature (cluster B); a tandem duplication signature and/or CDK12 biallelic inactivation (cluster C)	-	Nivolumab + ipilimumab for 4 cycles and nivolumab as maintenance (up to 1 year)	DCR	January 2026
IMPACT NCT03570619 Phase II	*n* = 40 Patients with metastatic cancers and CDK12 mutations: mCRPC (cohort A), metastatic solid tumors (non-prostate) (cohort B)	Patients must be ≥2 weeks from most recent systemic therapy or most recent radiation therapy	Nivolumab + ipilimumab for 4 cycles and nivolumab as maintenance (up to 1 year)	PSA50 ORR	September 2021
ImmunoProst trial NCT03040791 Phase II	*n* = 38 Patients with germline and somatic DRD (including HR and MMRd)	Documented prostate cancer progression, during treatment with docetaxel	Nivolumab	PSA response rate	January 2022
Neptunes NCT03061539 Phase II	*n* = 175 mCRPC patients with immunogenic biomarker positive disease (DRD–MMRd–high tumor-infiltrating lymphocyte)	1 or more lines of systemic treatment for mCRPC	Nivolumab + ipilimumab for 4 cycles and nivolumab as maintenance (up to 1 year)	PSA50 Radiological response conversion of CTC count	April 2022
NCT03248570 Phase II	*n* = 50 Patients with mCRPC with or without DNA damage repair defects	Patients must have received prior 2nd hormonal therapy (abiraterone, enzalutamide and/or apalutamide)	Pembrolizumab	rPFS	July 2023
NCT 04019964 Phase II	*n* = 15 Patients with at least one of the following genetic alterations: MMRd, MSIh, TMBh, inactivating mutation of CDK12	Prior local therapy with prostatectomy or EBRT/brachytherapy is required. Prior salvage or adjuvant radiation therapy is allowed but not mandated. Radiation therapy must have been completed for at least 6 months	Nivolumab	PSA50	January 2025
**ICI + TKI combination**					
NCT04159896 Phase II	*n* = 49	2nd generation hormonal agent (i.e., abiraterone acetate, enzalutamide) and chemotherapy (docetaxel and/or cabazitaxel)	ESK981 (pan-VEGFR/TIE2 TKI) + nivolumab	PSA50 AEs	March 2022
CONTACT-02 NCT04446117 Phase III	*n* = 580	One 2nd generation hormonal therapy (i.e., abiraterone, apalutamide, darolutamide, or enzalutamide) for mCSPC, M0CRPC, mCRPC	Experimental arm: atezolizumab + cabozantinib Control arm: abiraterone acetate/enzalutamide	PFS OS	March 2022
**New drugs**					
NCT04631601 Phase I–II	*n* = 159	-	Multiple experimental arm: acapatamab + enzalutamide; acapatamab + abiraterone; acapatamab + AMG404 (anti PD1); AMG 404 monotherapy; acapatamab monotherapy	DLTs TEAEs	November 2022
NCT03792841 Phase I	*n* = 288	Second-generation hormonal therapy (abiraterone, enzalutamide, and/or apalutamide) and 1–2 (or unfit/refuses) taxane regimens for mCRPC	Acapatamab ± pembrolizumab, etanercept, or a CP450 cocktail	DLTs TEAEs	December 2025
NCT04633252 Phase I–II	*n* = 86 Patients with mCSPC and mCRPC	Second-generation hormonal therapy (abiraterone, enzalutamide, apalutamide, or darolutamide) Must have not had progression while on docetaxel if given for mCSPC or within 3 months of completing docetaxel for mCSPC	Docetaxel + M9241 (tumor-targeting immunocytokine) Docetaxel + M9241 (tumor-targeting immunocytokine) + Bintrafusp alfa (M7824)	DLTs AEs PFS	December 2022
PRO-MERIT NCT04382898 Phase I–II	*n* = 130	2–3 lines of systemic therapy for mCSPC and mCRPC setting	W_pro1 (BNT112) Cemiplimab + W_pro1 (BNT112)	DLTs TEAEs ORR	July 2023
**Immune combination with standard therapies**					
Rad2Nivo NCT04109729 Phase Ib–II	*n* = 36	-	Nivolumab (up 2 years) + radium-233 (6 cycles)	Phase 1b: safety Phase 2: ctDNA reduction after 6 weeks	June 2022
Checkmate 7DX NCT04100018 Phase III	*n* = 984	1–2 s generation hormonal therapies (no ≥1 s generation hormonal therapy in the mCRPC setting)	Experimental arm: nivolumab + docetaxel Control arm: placebo + docetaxel	rPFS OS	April 2023

N: number of patients, MMD: mismatch repair deficiency, mCSPC: metastatic castration-sensitive prostate cancer, M0CRPC: non-metastatic castration-resistant prostate cancer, mCRPC: metastatic castration-resistant prostate cancer, nmBRPC non-metastatic biochemical recurrent prostate cancer, CP450: cytochrome P450, PSA50: prostate specific antigen decline ≥50%, ORR: objective response rate, NGS: next-generation sequencing, DCR: disease control rate, AEs: averse events, PFS: progression-free survival, OS: overall survival, DLTs: dose limiting toxicities, TEAEs: treatment-emergent adverse events, ctDNA: circulating tumor, rPFS: radiological progression-free survival.
